# The Gill Microbiota of *Argopecten purpuratus* Scallop Is Dominated by Symbiotic Campylobacterota and Upwelling Intensification Differentially Affects Their Abundance

**DOI:** 10.3390/microorganisms10122330

**Published:** 2022-11-25

**Authors:** Roxana González, Carlos Henríquez-Castillo, Karin B. Lohrmann, María Soledad Romero, Laura Ramajo, Paulina Schmitt, Katherina Brokordt

**Affiliations:** 1 Laboratorio de Fisiología y Genética Marina (FIGEMA), Departamento de Acuicultura, Facultad de Ciencias del Mar, Universidad Católica del Norte, Larrondo 1281, Coquimbo 1781421, Chile; 2 Centro de Estudios Avanzados en Zonas Áridas (CEAZA), Larrondo 1281, Coquimbo 1781421, Chile; 3 Departamento de Biología Marina, Facultad de Ciencias del Mar, Universidad Católica del Norte (UCN), Coquimbo 1781421, Chile; 4 Center for Climate and Resilience Research (CR)2, Santiago 8370449, Chile; 5 Grupo de Marcadores Inmunológicos, Laboratorio de Genética e Inmunología Molecular, Instituto de Biología, Pontificia Universidad Católica de Valparaíso, Valparaíso 2340000, Chile; 6 Centro de Innovación Acuícola (AquaPacífico), Universidad Católica del Norte, Larrondo 1281, Coquimbo 1781421, Chile

**Keywords:** microbiota, mollusks, Campylobacterota, symbiont, upwelling, climate change, scallop aquaculture

## Abstract

Despite the great importance of gills for bivalve mollusks (respiration, feeding, immunity), the microbiota associated with this tissue has barely been characterized in scallops. The scallop *Argopecten purpuratus* is an important economic resource that is cultivated in areas where coastal upwelling is intensifying by climate change, potentially affecting host-microbiota interactions. Thus, we first characterized the bacterial community present in gills from cultivated scallops (by 16S rRNA gene amplicon sequencing) and assessed their stability and functional potential in animals under farm and laboratory conditions. Results showed that under both conditions the gill bacterial community is dominated by the phylum Campylobacterota (57%), which displays a chemoautotrophic potential that could contribute to scallop nutrition. Within this phylum, two phylotypes, namely symbionts A and B, were the most abundant; being, respectively, taxonomically affiliated to symbionts with nutritional functions in mussel gills, and to uncultured bacteria present in coral mucus. Additionally, in situ hybridization and scanning electron microscopy analyses allowed us to detect these symbionts in the gills of *A. purpuratus*. Given that shifts in upwelling phenology can cause disturbances to ecosystems, affecting bacteria that provide beneficial functions to the host, we further assessed the changes in the abundance of the two symbionts (via qPCR) in response to a simulated upwelling intensification. The exposure to combined decreasing values in the temperature, pH, and oxygen levels (upwelling conditions) favored the dominance of symbiont B over symbiont A; suggesting that symbiont abundances are modulated by these environmental changes. Overall, results showed that changes in the main Campylobacterota phylotypes in response to upwelling intensification could affect its symbiotic function in *A. purpuratus* under future climate change scenarios. These results provide the first insight into understanding how scallop gill-microbial systems adapt and respond to climate change stressors, which could be critical for managing health, nutrition, and scallop aquaculture productivity.

## 1. Introduction

The microbiota is a complex and dynamic composition of commensal, symbiotic, and pathogenic microbes, including bacteria, archaea, fungi, protists, and viruses [1,2]. The diversity of microbial taxa comprising the microbiota can be classified into resident (autochthonous) and transient (allochthonous) populations. While the former is intimately associated with the host by colonizing epithelial and mucosal tissues, the appearance of non-resident free-living microbial taxa are highly influenced by environmental conditions [3]. Knowledge about the microbial communities associated with different host species has grown enormously in recent years [4]. Although there have been notable advances in understanding host-microbiota interactions about how each entity influences the other, major gaps remain in our knowledge of the assembly and dynamics of these intimate host-associated microbial communities [5]. Culture-independent assessment of microbial diversity, together with the use of gnotobiotic animals, has revealed the microbial contributions to the host [6]. Evidence indicates that the microbiota provides multiple benefits to the host, such as nutrition [7,8], protection against pathogens [9,10], and resistance to environmental changes [11]. In addition, under certain circumstances, the existing balance between the microbiota and its host is disrupted, causing dysbiotic states that negatively affect the host’s health [12].

The gills in bivalve mollusks display main functions such as respiration, capturing and transporting food particles, as well as being a mucosal barrier against potential pathogens [13,14]. In addition, this tissue can harbor chemoautotrophic bacteria, which fix carbon and/or inorganic nitrogen and provide their hosts with organic compounds [8]. Interestingly, recent studies showed that the association of bivalves with certain microorganisms can be crucial for adapting to diverse and challenging marine environments. For example, chemosymbiotic bacteria present in the gills of several bivalve species living from the intertidal zone to the hadal realm are fundamental for the nutrition and survival of the host [8]. Similarly, a new family of filamentous bacteria of the phylum Campylobacterota was described as a gill epibiont of mussels from the *Bathymodiolus* genus inhabiting several hydrothermal marine environments, and this association was proposed as a nascent stage of an endosymbiosis [15]. Campylobacterota (previously known as Epsilonproteobacteria) have been also found in the hemolymph of the scallop *Argopecten purpuratus* [16].

The scallop *A. purpuratus* is a bivalve mollusk of the family Pectinidae, which inhabits semi-enclosed bays from Sechura, Peru (6° S), to Valparaíso, Chile (33° S) [17], and is an important economic resource for both countries. This scallop inhabits the Humboldt Current System, one of the most productive Eastern Boundary Upwelling System (EBUS) worldwide [18]. These ecosystems are fertilized by deep nutrient-rich water which is also colder and presents low oxygen (O_2_) and high carbon dioxide (CO_2_) concentrations (low pH) that generate local cooling, deoxygenation, and acidification conditions at coastal habitats [19,20,21]. Climate change is affecting the upwelling phenology in some EBUS areas [19,22], and climate projections alert of upwelling events more intense, frequent, and longer in the future [19,23]. Changes in upwelling phenology may affect both natural marine populations and species subjected to aquaculture with important impacts on national and local economies and related socio-ecological systems [19,24], such as *A. purpuratus*. Therefore, it is urgent to advance in evaluating how cultured organisms are facing environmental conditions imposed by intense upwelling. 

The metabolic requirements of animals will be remodeled by global climatic change, and these shifts will be felt most acutely by animals that possess little control over their internal body condition [25], such as bivalve mollusks. Moreover, scallops are semi-open biological systems that filter large volumes of water (3–9 L/h/g dry mass) through their gills [26], so during most of their life, they are exposed to a great variety of environmental microorganisms and to diverse microbial communities that are part of their microbiota [5]. For these reasons, it is relevant to study the microbiota associated with this species, focusing on bacterial groups usually associated with hosts inhabiting “extreme” environments that provide beneficial functions to the host. 

Therefore, in the present study, we aimed to characterize the microbial community present in the gills of *A. purpuratus* by 16S rRNA gene amplicon sequencing from gDNA samples of cultivated individuals obtained directly from the farming area and from the same stock, but maintained under controlled laboratory conditions. By the comparison of the gill microbiota between scallops under these two conditions, we determined: (i) the number of bacterial groups which formed part of the resident gill microbiota and displayed a more stable association with scallops (namely the stability of specific bacterial groups); (ii) the microbial balance between the resident and transient microbiota, which is important because many of these bacteria could present antagonistic interactions with pathogenic bacterial groups present in low abundances in the microbiota; and (iii) the effect of maintaining scallops under laboratory conditions for culture purposes (e.g., reproductive conditioning for larval production) on the microbiota composition. Subsequently, molecular approaches (such as taxonomic analysis of the full 16S rRNA gene and in situ hybridization) were combined with electron microscopy to characterize two potential symbionts of the phylum Campylobacterota (because this was the most abundant in the gills). Finally, quantitative PCR was used to assess the changes in the abundance of the two symbionts in response to a simulated upwelling intensification, namely decrease in temperature, pH, and oxygen level. 

From this study, we aimed to contribute to the understanding about bivalve-microbiota interaction by: (i) generating new knowledge based on the composition, the stability and the potential function of the microbiota associated with gills of scallops; (ii) identifying and characterizing the main Campylobacterota phylotypes present in the gills; and (iii) determining the impact of upwelling intensification on the most abundant bacterial phyla that may be relevant to the host. Understanding how host-microbial systems adapt and respond to climate change stressors is critical for managing health, nutrition, and maximizing aquaculture productivity.

## 2. Materials and Methods

### 2.1. Scallop Maintenance, Experimental Procedure and Sample Collection

The study was conducted according to the guidelines of the Declaration of Helsinki, and approved by the Ethical Committee of the Universidad Católica del Norte (UCN) of Chile (protocol code: 02_2022).

Adult *Argopecten purpuratus* scallops (16 months of age, 60–70 mm shell height, *n* = 180) were sampled from the aquaculture facilities of the UCN located in the Tongoy Bay, Chile (30°16′ S, 71°35′ W). For comparison of gill microbiota between farm- and laboratory-kept scallops, a group of scallops was sampled directly in Tongoy Bay (Farm group; water temperature of 15–16 °C, 10 m depth) and another group from the same cultivated stock was transferred to the laboratory at the UCN in Coquimbo city (Laboratory group). In the laboratory, scallops were maintained for three weeks in 200 L tanks supplied with filtered (pore size 50 µm), aerated, running seawater (~16 °C), and fed daily with a diet composed by a mixture of 50% *Isochrysis galbana* and 50% *Nannochloris sp.* (6 × 10^6^ cells mL^−1^). 

For the upwelling intensification experiment, scallops were transferred to laboratory facilities at Tongoy Bay, and exposed to: (i) intense upwelling simulation for 16 days, which consisted in conditions of reduced temperature (10.83 ± 0.08 °C), pH (7.56 ± 0.02), and dissolve oxygen (4.22 ± 0.08 mL L^−1^); (ii) upwelling recovery simulation, where scallops after being exposed to the previous condition were exposed for 8 days to normal conditions of temperature (14.78 ± 0.01 °C), pH (8.00 ± 0.02), and dissolved oxygen (6.72 ± 0.11 mL L^−1^); and (iii) continuous absence of upwelling conditions (control: 15.0 ± 0.01 °C, pH 8.0 ± 0.02 and 7.0 ± 0.1 mL O_2_ L^−1^), where animals were maintained for 24 days.

During the experiment, the animals were fed the same diet described above. pH and DO treatments were achieved by aerating seawater with a mix of air, pure CO_2,_ and pure N_2_ gases using Mass Flow Controllers (Aalborg, Orangeburg, NY, USA). Water temperature was controlled using a chiller (BOYU, Model L075, Jiangsu, China). In order to maintain water quality and stable salinity levels at 34 ‰, aquaria were cleaned and refilled every 2 days with filtered and pre-treated seawater (10 μm plus UV-filter) with the same properties as experimental treatments tested. During the experiment, pH (NBS scale) discrete measurements were performed every day using a Metrohm 780 Meter (Metrohm^®^, Herisau, Switzerland) connected to a combined electrode (Aquatrode Plus with Pt1000, Metrohm^®^). Oxygen, salinity, and temperature conditions were also recorded every day for all experimental aquaria by using a Dissolved Oxygen meter (Model HI98193, HANNA^®^, Woonsocket, RI, USA) and a digital salinometer (Eutech^TM^, Eutech-Salt-6, Zurich, Switzerland), respectively. These experimental conditions are detailed in the Supplementary files (Figure S3). Gill samples were harvested from scallops and preserved in 100% ethanol for subsequent extraction of genomic DNA (gDNA). 

### 2.2. Genomic DNA Extraction and Deep Amplicon Sequencing of the 16S rRNA Gene 

Genomic DNA (gDNA) present in *A. purpuratus* gills was extracted with the Wizard Genomic DNA purification kit (Promega, Madison, WI, USA) according to the manufacturer’s instructions. The integrity, concentration, and purity of the gDNA were verified by 1% agarose gel, Qubit 3.0 (Life Technologies, Carlsbadm, CA, USA), and Epoch ™ microplate spectrophotometer (BioTek, Winooski, VT, USA), respectively. The 16S rRNA gene of bacterial communities was amplified and sequenced, targeting the variable regions V3–V4 (341F: 5′-CCTACGGGNGGCWGCAG-3′; 805R: 5′-159 GACTACHVGGGTATCTAATCC-3′ [27]) and using gDNA from 6 equimolar pool of gDNA (10 scallops by each pool) from each experimental condition. Paired-end multiplex sequencing (2 × 300 bp read length) was performed from individual samples by Macrogen Inc. (Seoul, Korea) on a MiSeq system (Illumina^®^, San Diego, CA, USA) using the MiSeq Reagent Kit v3 according to manufacturer’s instruction. Raw sequence data are available in the SRA database BioProject ID PRJNA844115. 

### 2.3. 16S rRNA Deep Amplicon Sequencing Analysis

All amplicon sequence data were analyzed together using the DADA2 package (v1.11.3) implemented in R (v3.4.4). The primers were removed using CutAdapt (v1.2.1) [28], and the sequences from each pair were trimmed to 210 and 190 bases, respectively, and quality filtered (truncLen = c (210, 190), maxEE = 1, maxN = 0, truncQ = 11, rm.phix = TRUE). Amplicon Sequence Variants (ASVs) were inferred from de-replicated sequences. Chimeras were removed using the “consensus” removal method. Taxonomic assignment was performed using the Silva v138.1 rRNA gene database. Subsequent analyses were performed using the microeco package in R environment [29]. Briefly, the relative abundance of ASVs in each sample and community analysis (observed composition, alpha-diversity, and beta/diversity indexes) were calculated from subsampled datasets with the phyloseq package [30]. Modules were inferred from a correlation network [31]. For this, we used the SparCC, that estimates the linear Pearson correlations between the log-transformed compositional data. Module-specific and by sample functional profiles were computed using FAPROTAX [32]. Linear discriminant analysis (LDA) effect size (LEfSe) algorithm [33] was used to identify differentially represented functional traits between conditions. All the functional analyses were performed with microeco v 0.12.0 [29]. Alpha diversity was evaluated using Pielou’s evenness, Richness and Inverse Simpson index. Differences in alpha diversity between groups were analyzed using the Pair-wise ANOVA. A principal coordinate analysis was performed to visualize beta diversity, with PERMANOVA used in the differential test of distances among groups.

### 2.4. Phylogenetic Inference for the 16S rRNA Sequences from Campylobacterota

Full 16S rRNA gene sequences belonging to the Phylum Campylobacterota were extracted from samples obtained from *A. purpuratus* gills (bioproject ID PRJNA802391). We used BLAST to determine the percent identity of the short amplicon sequences analyzed in this work with respect to the full-length sequences. For this, a BLAST database was constructed with 58 ASVs using the blast+ tool. Short sequences were locally BLASTed using MEGABLAST (-max_target_seqs 5 -evalue 0.001 -outfmt 6 -task megablast). For the phylogenetic inferences, sequences were aligned using MAFFTv7 [34] with the G-INS-i refinement method and the phylogenetic tree was constructed using IQTREE2 [35] with ultrafast bootstrap (options -m TEST -bb 1000 -alrt 1000). The best-fit model, TVMe + I + G4, was chosen according to the Bayesian Informative Criteria (BIC).

### 2.5. In Situ Hybridization (ISH)

Embedded gill tissues in paraffin blocks were sectioned and placed on silane-treated slides (Merck Life Science, Gillingham, UK) for in situ hybridization (ISH) analysis. Two digoxigenin (DIG)-labelled DNA probe (654 bp of the Symbiont A and 491 pb of Symbiont-B) 16S rRNA gene was generated by PCR using DIG-labelled dNTPs (Merck Life Science, Darmstadt, Germany) and specific primers (Supplementary files Table S1). ISH assays were carried out following standard protocols [36]. Briefly, tissues were permeabilized with Proteinase K (100 µg mL^−1^) (Merck Life Science, Darmstadt, Germany) for 30 min at 37 °C in a moisture chamber. The DIG-labelled probe was diluted 1:10 in hybridization buffer (60% formamide, 10% dextran sulfate, 2× saline sodium citrate buffer (SSC), and 0.2 µg µL^−1^ salmon sperm DNA) and denatured at 95 ◦C for 5 min before hybridization, overnight at 42 °C. Two post- hybridization washes containing 2× SSC, 6 M urea, and 0.2% bovine serum albumin (BSA) were performed at 42 °C, for 15 min each. Tissue sections were blocked with 6% skimmed milk and incubated with an anti-DIG monoclonal antibody conjugated to alkaline phosphatase (Merck Life Science, Darmstadt, Germany), for 1 h. The hybridization signal was revealed using NBT/BCIP and nuclei counterstained using nuclear fast red stain (Merck Life Science, Darmstadt, Germany). 

### 2.6. Scanning Electron Microscopy (SEM) 

Gill pieces were fixed with 1G4F [1% glutaraldehyde and 4% formaldehyde in sea water [37] and prepared for SEM. Gill pieces were dewaxed via three changes of xylene, passed through three changes of 100% ethanol, critical point dried using CO_2_, and ion sputtered with gold. Processed samples were viewed in a Hitachi SU 3500 scanning electron microscope (Tokyo, Japan), and images were saved. Measurements of filamentous bacteria were recorded directly with the SEM.

### 2.7. Absolute Quantification of Bacterial Symbionts by qPCR 

The 16S rRNA gene of symbiont A and B was amplified by PCR using the 16S rRNA specific primers (Supplementary files Table S1). The 16S rRNA fragments were cloned into the pGEM-T Easy vector (Promega, Madison, WI, USA) following manufacturer’s instructions. Subsequently, competent cells of *Escherichia coli* DH5-Alpha (Thermo Fisher, Walthamm, MA, USA) were transformed with the plasmid by heat shock and then cultured in agar plates LB/Amp/IPTG/X-gal overnight at 37 °C. Plasmids containing the corresponding insert were purified with an UltraClean^®^15 DNA Purification Kit (Mo Bio Laboratories, Carlsbad, NM, USA). Specific cloning was verified by plasmid sequencing. Then, seven-serial dilutions of each plasmid, ranging from 10^10^ to 10^3^ plasmid copies/µL were prepared to construct standard curves and to obtain absolute quantifications of the 16S rRNA gene copy number of each bacterial group by qPCR. Plasmid copies were calculated by the following formula: Number of copies/µL = 6.022 × 10^23^ (molecules/mole) × DNA concentrations (g/µL)/Number of bases pairs × 660 Da, were 6.022 × 10^23^ (molecules/mole) is Avogadro´s number and 660 Da is the average weight for a single base pair.

Quantitative PCR assays were performed in triplicate on a Stratagene Mx3005p Real Time PCR System thermocycler (Agilent Technologies, Santa Clara, CA, USA), using a final reaction volume of 20 µL composed of: 10 µL of Takyon ™ Rox SYBR^®^ MasterMix dTTP Blue (Eurogentec, Seraing, Belgium), 0.6 µL of each primer (10 mmol L^−1^) and 2 µL of plasmid DNA. The amplification program consisted of an initial denaturation at 10 min at 95 °C, followed by 40 cycles of 30 s at 95 °C and 1 min at 60 °C and dissociation curve detection. Standard curves were obtained by plotting the threshold cycle (C_q_) on the Y-axis and the natural log of concentration (copies/ µL) on the X-axis. We considered the following criteria: PCR efficiency (95–110%) and correlation coefficient R^2^ (0.99), which validates the linear relation between the threshold cycle and the natural log of concentration (copies/µL). Next, 100 ng of gDNA from scallop gill was analyzed by qPCR using the same settings. The copy numbers of 16S rRNA gene of each bacterial group in the sample were obtained by relating the C_q_ value to the respective standard curve. Differences in the abundance of the bacterial symbionts between treatments were verified with Mann-Whitney test (*p* < 0.05).

## 3. Results and Discussion

### 3.1. The Gill Microbiota of A. purpuratus Is Dominated by the Phylum Campylobacterota in the Farm and under Controlled Laboratory Conditions

The analyses of 16S rRNA gene amplicon sequences from gDNA samples of individuals obtained from the farm and from the laboratory, showed that of the 39 phyla identified, 51% (20 phyla) were present in both the farm and laboratory samples (Supplementary files, Figure S1A). The phylum Campylobacterota were the most abundant bacterial taxa in all analyzed samples from both environments, comprising 57% of the phyla present in gills, followed by the Proteobacteria (21.3%), Firmicutes (11.01%), and Bacteroidota (5.38%) (Supplementary files, Figure S1B). Some of these phyla have been described also as predominant bacterial groups in other scallops. For example, Firmicutes and Bacteroidota are dominant phyla in the gill of the scallop *Patinopecten yessoensis* [38]. In terms of alpha diversity, the scallops maintained under controlled laboratory conditions displayed a more diverse bacterial community compared to scallops from the farm (Supplementary files, Figure S2A) (Inv. Simpson index; *p* < 0.05). 

**Figure 1:**
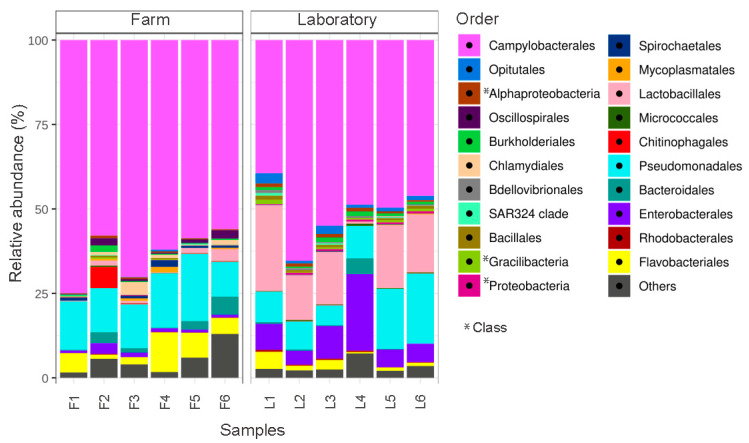
Comparison of relative abundances of bacterial groups in *Argopecten purpuratus* gills between scallops maintained under farm and laboratory conditions. Stack bar graphs indicate the average relative abundance of major bacterial orders found in the farm and laboratory-maintained scallops (*n* = 10 pooled individuals per sample).

Significant differences, in terms of community composition, were found between farm and laboratory individuals (Supplementary files, Figure S2B, Manova Pr (>F) = 0.001). Among the most significant differences, a notable increase in bacteria belonging to the phylum Firmicutes were detected in individuals under laboratory condition (from 0.01% in the farm to 12.3% in the laboratory), specifically in bacteria from the order *Lactobacillales* (genus *Enterococcus*) (Figure 1, Supplementary files, Figure S3). Bacteria from the *Enterococcus* genus have been used as probiotics in humans and marine invertebrates [39,40], although many species of this group can also be pathogenic [41]. Similarly, an increase in bacteria classified as *Enterobacteriaceae* (genus *Escherichia-Shigella*) were detected in the laboratory (4.4%) compared to the farm scallop (0.9%). The Gram-negative bacterium from the genus *Escherichia-Shigella* are potentially pathogenic, causing bacillary dysentery in humans (Shigellosis) [42]; this has also been observed in bivalves and even in fish, as part of the resident microbiota [43]. On the other hand, a decrease in bacteria from the phylum Bacteroidota under laboratory conditions was observed, in particular those of the order *Flavobacteriales* (genus *Winogradskyella)* from 4.5% in farm to 0.7% under laboratory conditions (Supplementary files, Figure S3). The genus *Winogradskyella* is commonly found in numerous marine organisms [44,45], including marine bivalves [9,16]. Interestingly, the genus *Winogradskyella* is a consistent member across most ostreid herpesvirus-1 microvariant (OsHV-1 µvar) resistant oyster families [9]. In *A. purpuratus*, *Winogradskyella* is the most abundant genus of the hemolymph [16]. However, it is uncertain what function(s) the *Winogradskyella* species could play in bivalve mollusks. In agreement with these results, differentially represented functional traits between conditions analysis (see Section 3.2 for details), showed differences in the main functional profiles between farm and laboratory condition; the pathogenic functions being most pronounced in scallops under laboratory condition (Supplementary files, Figure S4). 

Since some microbiota members may affect the host’s susceptibility to other bacteria [46,47], the effect generated by the observed loss or incorporation of certain bacteria from/to *A. purpuratus* gill microbiota (e.g., under laboratory conditions) remains to be elucidated. In general, it is known that excluding specific bacteria in a host can create space for other (previously excluded, i.e., niche opportunity) competitors in some biological systems [48]. In summary, when cultivated scallops were conditioned under laboratory environments, important changes in the abundance of groups with symbiotic and pathogenic potential, as well as in the functional profiles, are observed. The effects that this may have on the host’s physiology remain to be studied. 

### 3.2. The Gill Microbiota of A. purpuratus Have a Potential Chemoautotrophic Function That May Contribute to Its Nutrition

**Figure 2:**
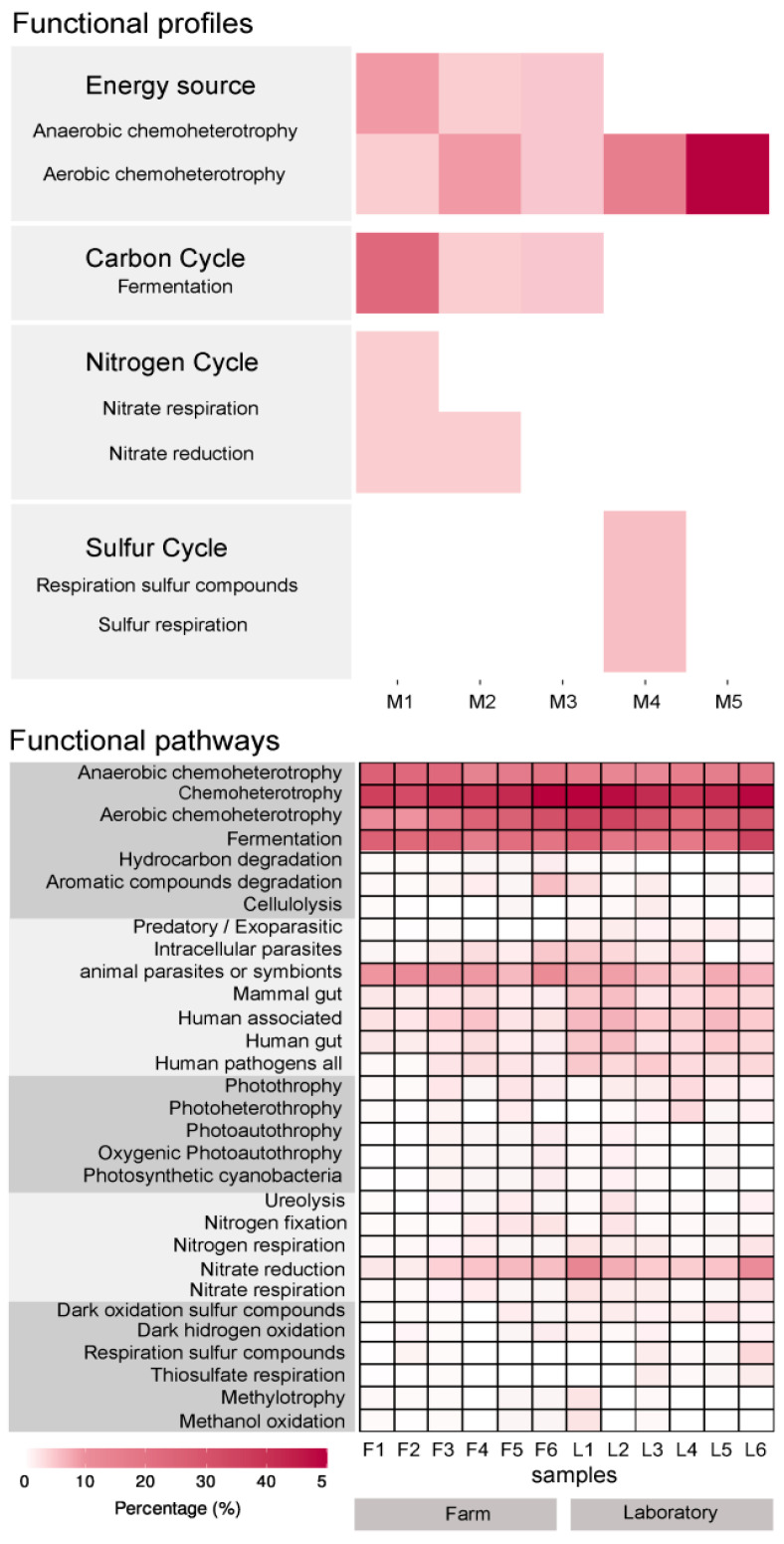
Predictive functional profiles based on 16S rRNA gene sequences from *Argopecten purpuratus* gills bacterial community (*n* = 10 pooled individuals per sample). Five bacterial modules of co-occurrence were identified (M). Heatmap of the most abundant functional pathways obtained from FUNPROTAX analysis for gill microbiota between farm and laboratory-maintained scallops. Abundance percentage is represented in the color temperature bar.

Based on co-occurrence analysis and functional profiling, we focused on the four main biological functions of the microbial community present in the gills of *A. purpuratus* (Figure 2). The main identified functions were: (i) obtaining energy by chemoautotrophic mechanisms, which is represented in all the identified bacterial modules of co-occurrence; (ii) carbon cycle, represented in four of the five bacterial modules; (iii) nitrogen cycle, represented in one bacterial module; and (iv) the sulfur cycle, represented in one bacterial module (Figure 2). The most abundant group in the gill microbiota corresponded to the phylum Campylobacterota and shares biological functions associated with energy production and carbon cycling. In this sense, members of the phylum Campylobacterota have been identified as ecto—or endosymbionts in species of bivalves, shrimps, crabs, and polychaetes, that inhabit hydrothermal environments [49,50]. Campylobacterota are chemoautotrophic microorganisms that can fix carbon through the reductive Tricarboxylic Acid Cycle (rTCA) cycle or, in some cases, by the Calvin–Benson–Bassham (CBB) [49]. Campylobacterota are commonly detected in low-oxygen and high-sulfide environments (van der Stel and Wösten, 2019), which is consistent with the functional potential found in *A. purpuratus* gills community enriched in anaerobic metabolisms. Recently, fermentation and anaerobic consumption of fermentation products (mainly acetate) oxidized by a variety of electron acceptors including NO_3_^−^, Fe(OH)_3_, and SO_4_^−2^ have been evidenced in low oxygen waters of the Humboldt Current System, with acetate as the major energy substrate for sulfate-reducing bacteria [51]. These environmental bacteria thus represent an important pool of genetic and functional diversity for the gill microbiota of *A. purpuratus*. While the nature of the association of Campylobacterota with the gills of marine bivalves is still unclear, it has been proposed that they could transfer nutrients to the host by diffusion of small molecules, or by endocytosis and intracellular digestion of the bacteria [52]. Our result highlights the dominance of Campylobacterota in the gill microbiota of *A. purpuratus* and confirms its potential role in the host nutrition using anaerobic metabolism, which could be relevant in the capacity of this scallop to adapt to low oxygen waters. In agreement with the taxonomic signatures, the presence of pathogenic traits was significantly more represented in the laboratory than in the farm condition (Supplementary files, Figure S4). 

### 3.3. The Campylobacterota from Scallops Are Part of a Widely Distributed Family of Symbionts of Bivalves That Inhabit Extreme and Challenging Environments

We extracted the full 16S rRNA gene sequences of the phylum Campylobacterota, corresponding to 58 ASVs that matched to the Campylobacterota detected here (100% perc. id. using BLAST). The two most abundant ASVs were named symbiont A and symbiont B. The closest relative to symbiont A corresponded to an uncultured Campylobacterota from the *Bathymodiolus* mussel gills (98% identity). In the case of symbiont B, the closest relative was an uncultured Campylobacterota bacterium present in coral mucus from the southern Caribbean (96% identity, accession number KU243328).

**Figure 3:**
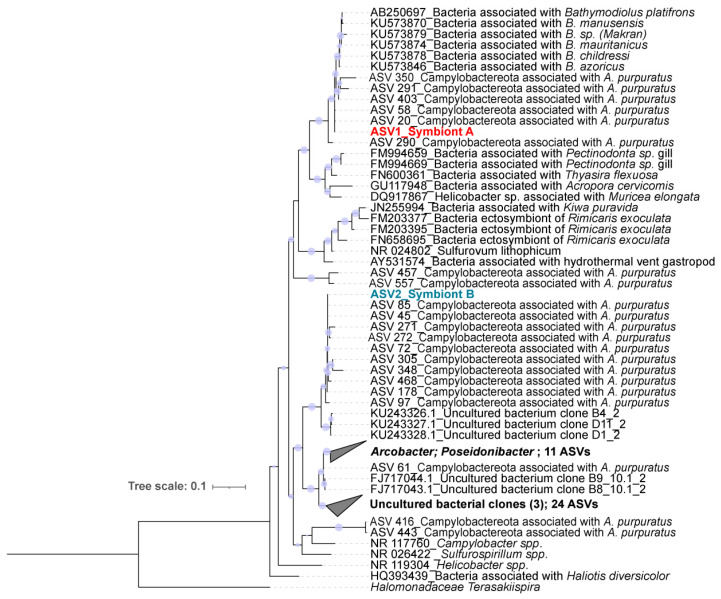
Condensed maximum-likelihood tree reconstruction of Campylobacterota based on the 16S rRNA gene. ML topology shown with SH-like approximate likelihood ratio support values (*n* = 1000) given at each node (values > 50% are shown). *Halomonadaceae terasakiispira* was used as root for the tree. The scale bar represents 10% estimated sequence divergence per nucleotide position.

Phylogenetic analysis showed that the 16S rRNA gene sequences of symbionts A and B are located at distant positions, each within a defined clade (Figure 3). The symbiont A sequence was taxonomically associated to Campylobacterota sequences from marine bivalve gills. This clade is a sister group of uncultured and unclassified bacterial sequences from invertebrates inhabiting deep-sea hydrothermal vents, such as bivalves (FN600361), gastropods (FM994656), and corals (DQ917867 and GU117971) (Figure 3). Therefore, the symbiont A is part of a recently identified family of epibionts Campylobacterota from *Bathymodiolus* mussels inhabiting distinct world areas [15].

### 3.4. Campylobacterota Are Located in the Surface of Scallop Gills

**Figure 4:**
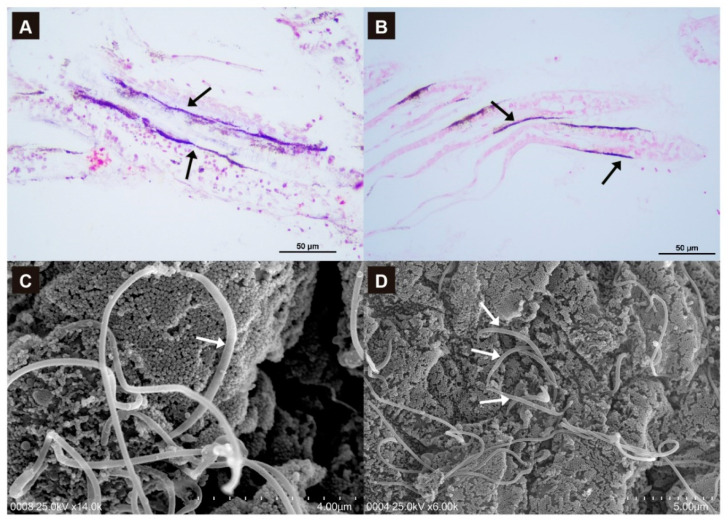
Localization of Campylobacterota in *Argopecten purpuratus* gills. In situ hybridization of symbiont-A (**A**) and -B (**B**) in scallop gill tissue. The labeling is observed microscopically as dark blue staining (arrows). (**C**,**D**). Scanning electron microscopy of scallop gills filament. Filamentous bacteria are observed (arrows).

We performed in situ hybridization (ISH) and Scanning Electron Microscopy (SEM) to detect and localize Campylobacterota in scallop gills. We designed two specific probes: SymA and SymB to target the 16S rRNA sequences of these symbionts. The presence of Campylobacterota DNA in the gills was confirmed by ISH in samples confirmed positive by sequencing (Figure 4A,B).

SEM images revealed the presence of filamentous bacteria in gill sections of *A. purpuratus*, however, as in other studies it was difficult to distinguish them from the host cilia [15]. Still, some distinctive features in thickness and length were identified, where potential Campylobacterota shows thicker and shorter structure in comparison with gill cilia (Figure 4C,D).

### 3.5. Upwelling Intensification Differentially Affects the Abundance of the Most Relevant Campylobacterota

Because the scallop *A. purpuratus* usually inhabits areas where coastal upwelling is intensifying [53], we assessed the effect of this condition on the abundance of Campylobacterota symbionts A and B in scallops exposed to an experiment simulating situations of absence (control), intensification, and recovery (similar to control) from upwelling events (Supplementary files, Figure S5). 

**Figure 5:**
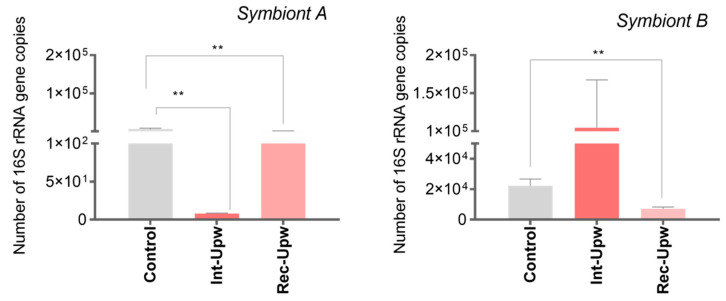
Abundance of Campylobacterota symbionts A and B in scallop gills exposed to upwelling intensification. Abundances are expressed as number of bacterial 16S rRNA gene copies in scallop gills exposed to upwelling intensification (Int-Upw: 10.83 ± 0.08 °C, pH 7.56 ± 0.02, 4.22 ± 0.08 mL O_2_ L^−1^) and recovery (Rec-Upw: 14.78 ± 0.01 °C, pH 8.0 ± 0.02, 6.72 ± 0.11 mL O_2_ L^−1^). Values are represented as the mean ± SE and asterisks indicate significant differences compared with control scallops (absence of upwelling—Control: 15.0 ± 0.01 °C, pH 8.0 ± 0.02 and 7.0 ± 0.1 mL O_2_ L^−1^) (** *p* < 0.005).

The results showed opposite dynamics between the two bacterial symbionts (Figure 5). Symbiont A showed a significant decrease in abundance under the intensified upwelling condition, respect to control and recovery conditions (by, respectively, 99 and 89%) (*p* < 0.05); whereas symbiont B showed a tendency to increase in abundance under the intensified upwelling condition and decreased after recovery from upwelling (Figure 5). Several studies show that different members from the Campylobacterota may exhibit different adaptation strategies to changes in their environment, and these may depend on the mechanisms that regulate their metabolic pathways [54,55]. In general, the mechanisms that regulate the metabolic pathways of the Campylobacterota are not fully understood, and the response to environmental change in host-associated bacteria is uncertain [56]. However, it has been determined that one of the most important strategies in regulating the electron transport chain in some Campylobacterota is the ladder mechanism. Here, the absence of the preferred electron acceptor causes all other reductases to be expressed, regardless of their cognate substrates; this suggests a beneficial evolutionary strategy to adapt to the electron acceptors available in the environment efficiently [56]. Nevertheless, in dynamic environments, where respiratory substrates are temporarily available, fast-growth might be the best strategy to gain an advantage over other competing microorganisms [57], which could cause an increase in the abundance of one bacterial symbiont over another. It is possible that two symbionts within the same group of Campylobacterota show different flexibility and adaptation strategies to environmental changes.

On the other hand, the host´s effect on the relative composition of its microbiota has been demonstrated for *A. purpuratus* [16,58]. Specifically, it has been shown that the expression level of some immune response effectors modulates the abundance of bacterial groups present in the hemolymph of this scallop [16,58]. The climate changes constitute a gradually increasing and persistent stress with an overall negative effect on host immunity [59]. Thus, the overall consequences of climate change for the invertebrates will depend in part on the interaction between the immune response and the host microbiota

Many host-associated Campylobacterota show a loss of genes that regulate metabolic pathways, which would help them adapting to changes in environmental oxygen levels, so their responses are often more uncertain relative to free-living Campylobacterota [56]. In this context, we observed important changes in the abundance of two dominant symbionts in gills that could be relevant to the host and impact its physiology. After upwelling intensification conditions, we specifically observed a decrease in the abundance of the symbiont A, closely related to gill epibionts that have evolved with other bivalves to supply nutritional functions. This symbiont may have a closer association with *A. purpuratus* and a lower adaptive capacity to the assessed climate change scenario.

In the context of climate change, marine bivalves will be exposed to stressful conditions that could harm their health. The microbiota is relevant to the health of organisms, providing benefits when it is in balance or affecting them negatively when this balance is disrupted. Emerging evidence in the field of medicine suggests that a growing number of human diseases result from a microbiome imbalance (or dysbiosis), questioning the traditional view of a singular pathogenic agent [60]. Polymicrobial infections in marine environments affecting bivalves under culture have just begun to be deciphered [61,62], and more of them involving environmental changes could arise in the future as several mass mortalities in marine bivalves have no evident etiological agent. It is possible that many diseases seen in marine systems are the result of microbial dysbiosis and the rise of opportunistic or polymicrobial infections [63]. For these reasons, a multidisciplinary approach that addresses the questions of microbial symbiosis in both healthy and diseased states, as well as integrative studies that consider environmental impacts on microbiota-host interactions, will be mandatory for aquaculture viability in the future.

## 4. Conclusions

The analysis of the gill microbiota of *A. purpuratus* indicates a predominance of the phylum Campylobacterota and a mainly chemoautotrophic microbial community function. Furthermore, we observed that, despite the stability in the dominance of the Campylobacterota, the gill microbiota changes under laboratory conditions, and as a result, some bacterial groups with pathogenic potential emerge. The most representative members of the gill Campylobacterota of *A. purpuratus* are related to gill symbiont bacteria of marine bivalves inhabiting challenging environments. The dynamics of these symbionts in response to upwelling intensification showed a differential abundance pattern. This study demonstrates that the gill microbiota is dynamic to some extent, and that some of the putative functions associated with crucial bacterial groups could be affected in the scenario of climate change.

## Data Availability

Raw sequence data are available in the SRA database BioProject ID PRJNA844115 and BioProject ID PRJNA802391.

## Supplementary Materials

The following supporting information can be downloaded at: https://www.mdpi.com/article/10.3390/microorganisms10122330/s1. Figure S1: (A). Venn diagrams showing the number of common and distinct bacterial phylum in gills between farm and laboratory (Lab) maintained *Argopecten purpuratus* scallop. (B). Comparison of relative abundance of bacterial phylum in gills between scallops maintained under farm and laboratory conditions. Figure S2: Comparison of diversity in gills between farm and laboratory (Lab) maintained *Argopecten purpuratus* scallop. Alpha diversity indices (Pielou’s evenness (A), Richness (B) and Inverse Simpson (C) indices) found in gill bacterial microbiota from analyzed scallops. Pair-wise ANOVA of diversity measures between groups (Laboratory and farm) was computed. The asterisk represents the significance in the ANOVA (* Pr (>F) < 0.001). (D). Beta diversity analysis of the farm and laboratory-maintained scallop gill microbiota by Principal Coordinate Analysis (PCoA). Figure S3: Heat map showing the abundances of different taxa, using the lowest taxonomic level available, in gills of *Argopecten purpuratus* cultivated scallops maintained in the farm and laboratory (Lab). Figure S4: Differential representation analysis plots of functional biomarkers identified in gill bacterial microbiota of *Argopecten purpuratus*. Linear Discriminant Analysis (LDA) Effect Size (LEfSe) was used for the discovery of functional differences between cultivated scallops maintained under laboratory and farm conditions. The left panel shows the LDA scores of the top 10 differentially represented functional traits. The threshold for the logarithmic discriminant analysis (LDA) score was 4. The right panel shows the relative abundances of these functions and the significance of the differences. * *p* < 0.05; ** *p* < 0.001. Figure S5: Schematic representation of upwelling experiment performed in the present study. Experimental variables (data are means ± SD), sampling time points, and the number of biological replicates sampled at each time point are indicated in three experimental replicates per condition. Table S1: Oligonucleotide primers used for in situ hybridization (ISH) and gene expression analysis of bacteria Campylobacterota of *A. purpuratus.*
