# Supplementary material for: The Gill Microbiota of Argopecten purpuratus Scallop Is Dominated by Symbiotic Campylobacterota and Upwelling Intensification Differentially Affects Their Abundance
Source: Microorganisms. 2022 Nov 25;10(12):2330. doi: 10.3390/microorganisms10122330 (PMC9781997; doi:10.3390/microorganisms10122330)
Supplement: Supplementary file 1 [file microorganisms-10-02330-s001.zip › microorganisms-2006177-supplementary.pdf]

Supplementary File

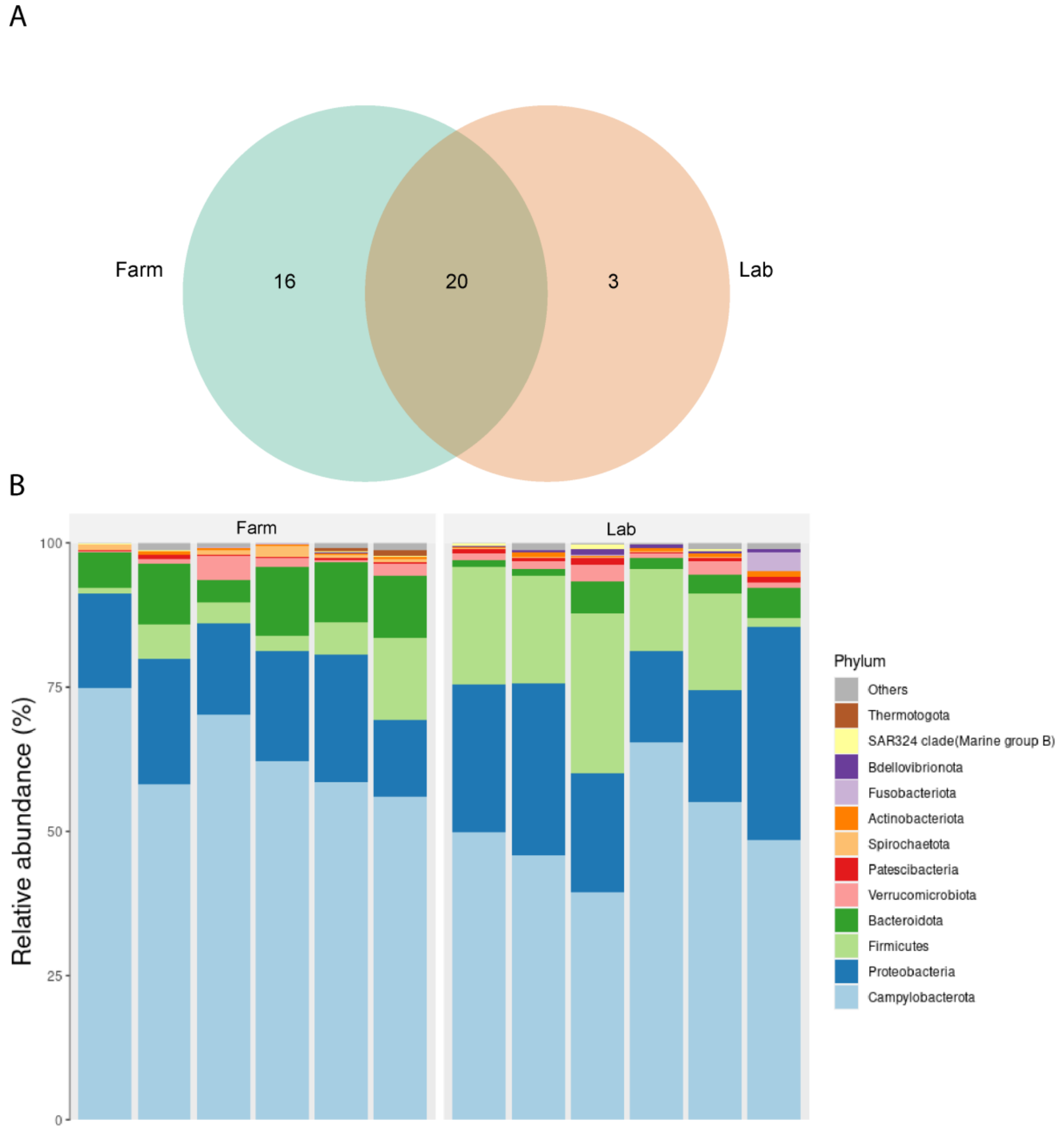

Figure S1. (**A**). Venn diagrams showing the number of common and distinct bacterial phylum in gills between farm and laboratory (Lab) maintained *Argopecten purpuratus* scallop. (**B**). Comparison of relative abundance of bacterial phylum in gills between scallops maintained under farm and laboratory conditions.

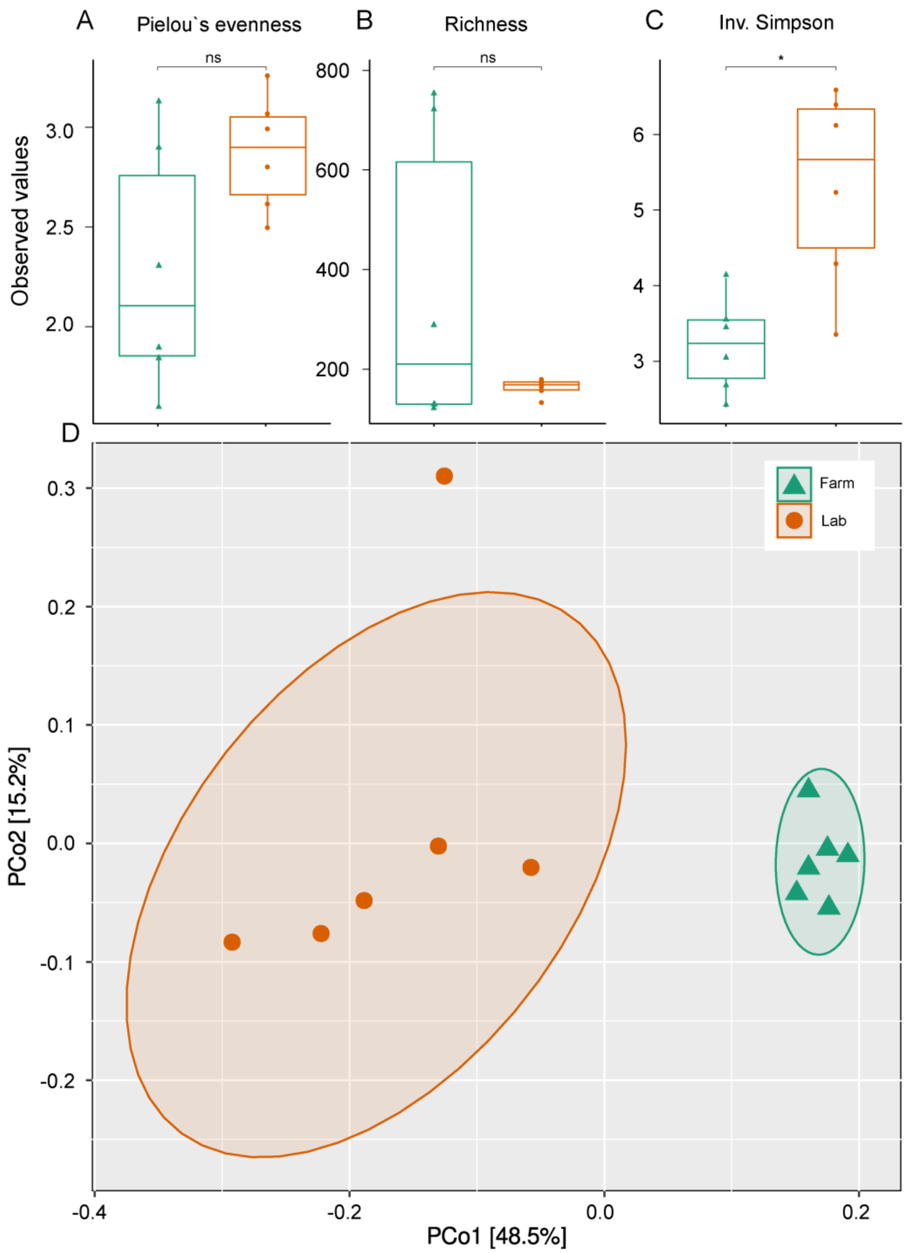

Figure S2. Comparison of diversity in gills between farm and laboratory (Lab) maintained *Argopecten purpuratus* scallop. Alpha diversity indices (Pielou's evenness (**A**), Richness (**B**) and Inverse Simpson (**C**) indices) found in gill bacterial microbiota from analyzed scallops. Pair-wise ANOVA of diversity measures between groups (Laboratory and farm) was computed. The asterisk represents the significance in the ANOVA (* Pr (>F) < 0.001). (**D**). Beta diversity analysis of the farm and laboratory-maintained scallop gill microbiota by Principal Coordinate Analysis (PCoA).

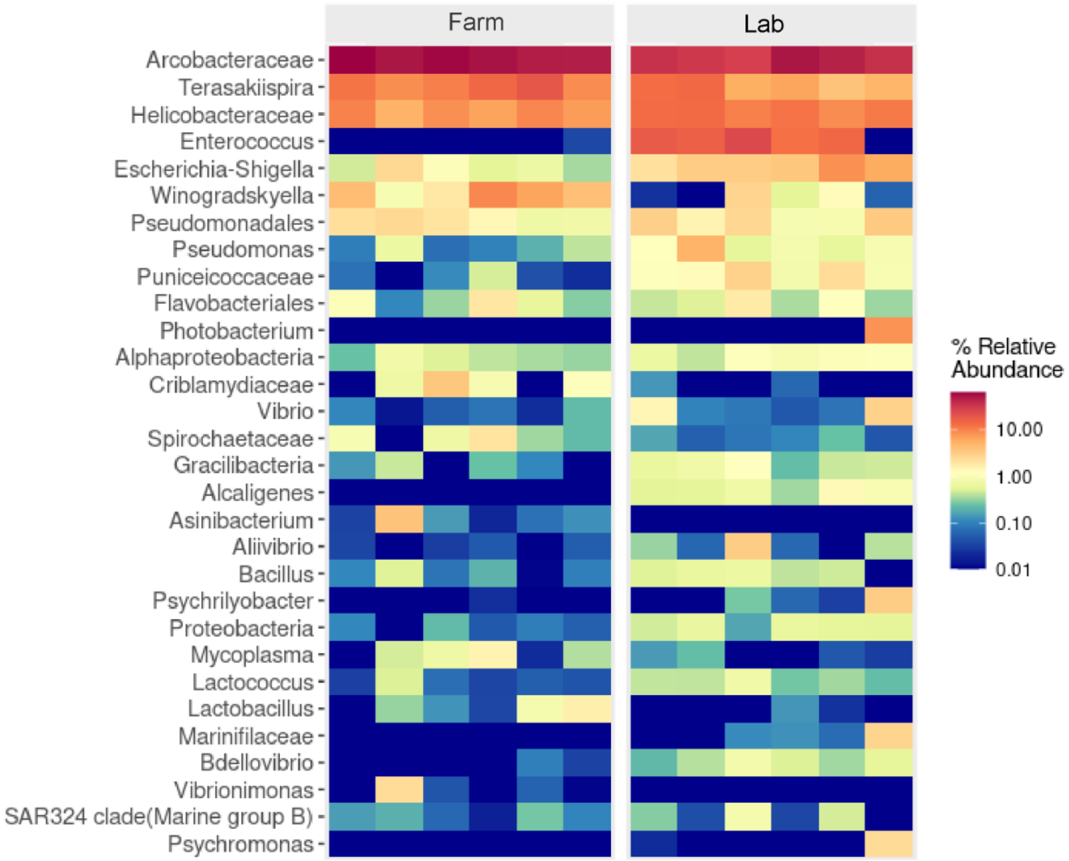

Figure S3. Heat map showing the abundances of different taxa, using the lowest taxonomic level available, in gills of *Argopecten purpuratus* cultivated scallops maintained in the farm and laboratory (Lab).

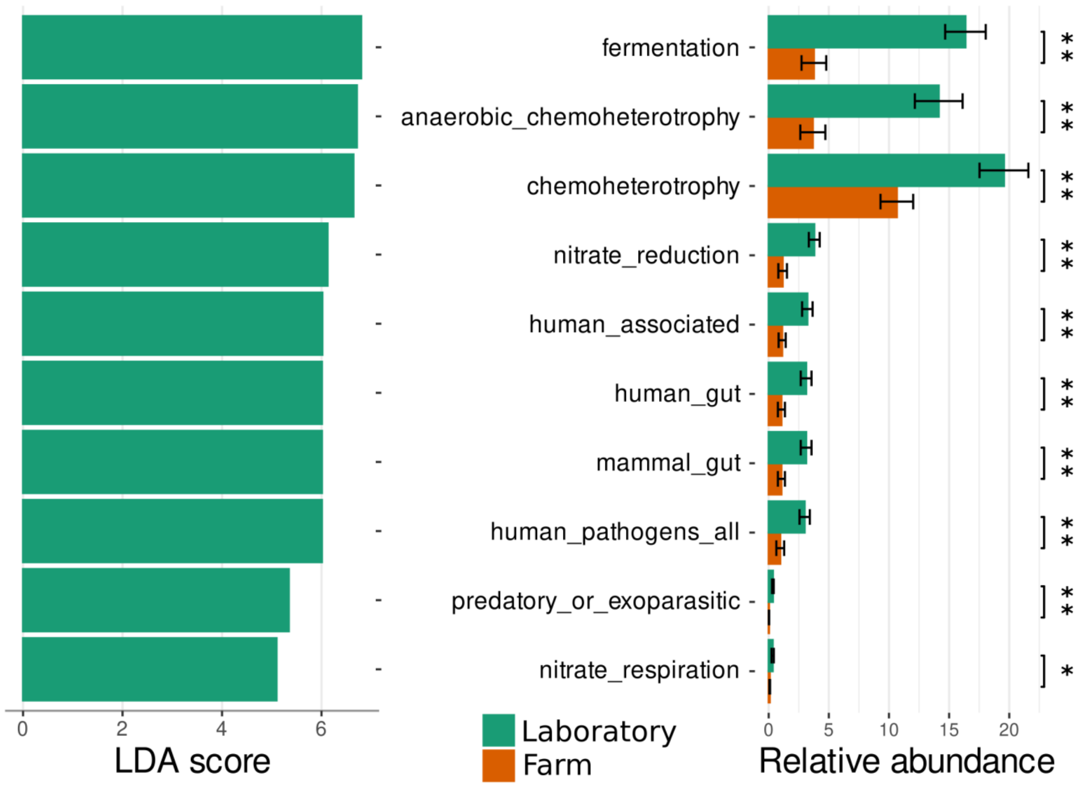

Figure S4. Differential representation analysis plots of functional biomarkers identified in gill bacterial microbiota of *Argopecten purpuratus*. Linear Discriminant Analysis (LDA) Effect Size (LEfSe) was used for the discovery of functional differences between cultivated scallops maintained under laboratory and farm conditions. The left panel shows the LDA scores of the top 10 differentially represented functional traits. The threshold for the logarithmic discriminant analysis (LDA) score was 4. The right panel shows the relative abundances of these functions and the significance of the differences. * $p < 0.05$; ** $p < 0.001$.

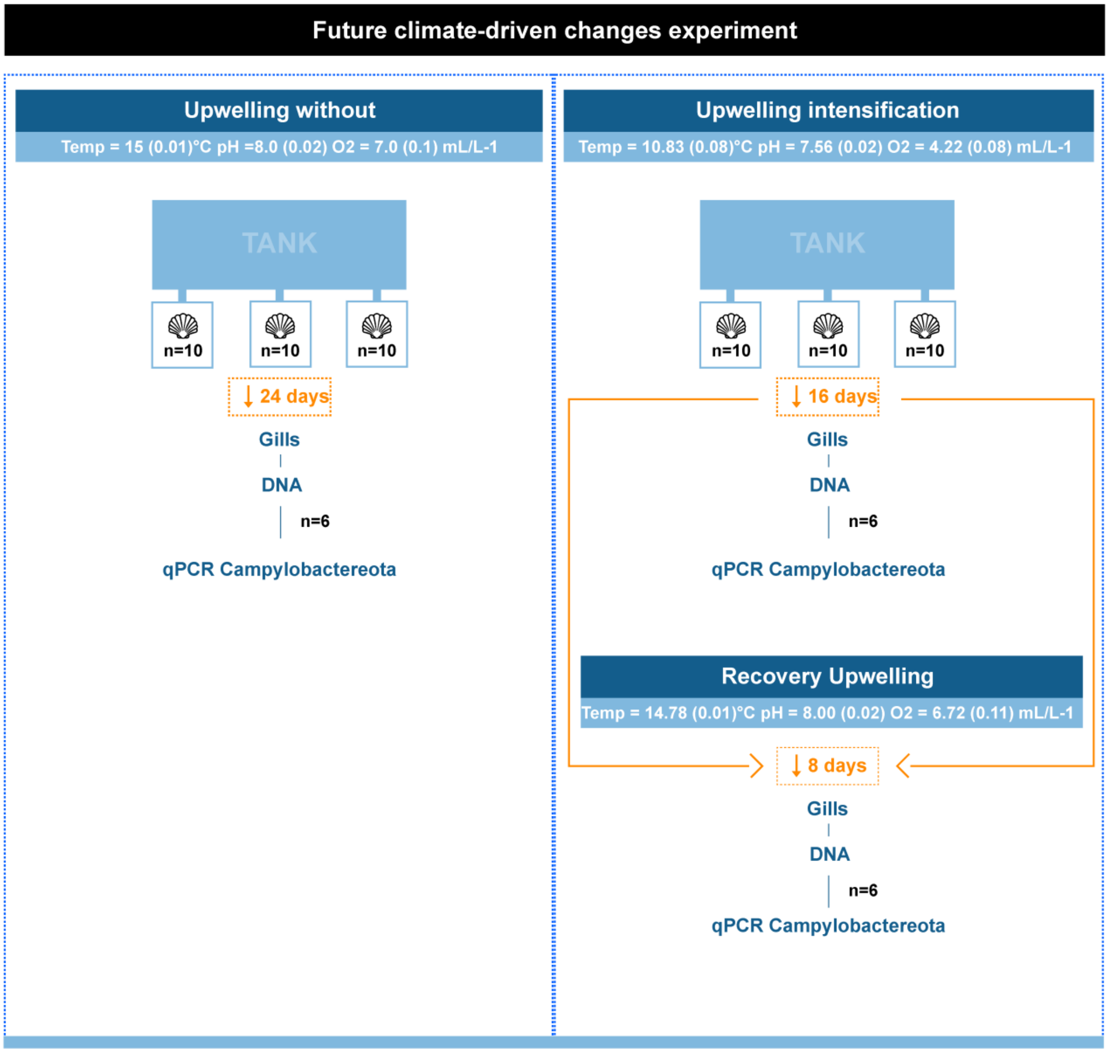

Figure S5. Schematic representation of upwelling experiment performed in the present study. Experimental variables (data are means ± SD), sampling time points, and the number of biological replicates sampled at each time point are indicated in three experimental replicates per condition.

Table S1. Oligonucleotide primers used for *in situ* hybridization (ISH) and gene expression analysis of bacteria Campylobacterota of *A. purpuratus*

Primer name	Forward primer (5′-3′)	Reverse primer (5′-3′)
**Primers for ISH**		
SymA	CAAGTCGAACGAGAACGGGA	AGTCGCCTTCGCTTTTGGTA
SymB	TGGGGTTGTATAGCATCAGC	TTCCAGTAGATCGCCTTCGC
**Primers for qPCR**		
qSymA	CCTTCTAGCAGGGGATAACATCG	GGACCGTGTCTCAGTTCCAGT
qSymB	GCATCAGCTTGTTGGTGGGG	CGTCAGGCTTTCGCCCATTG
